# Amoxicillin-induced bullous erythema multiforme: a case report

**DOI:** 10.1097/MS9.0000000000001513

**Published:** 2023-11-16

**Authors:** Seema Sitaula, Bikash K. Shah, Sanjeev Kharel, Alisha Yadav, Nahakul Poudel, Bhusan Shrestha

**Affiliations:** a Department of Dermatology and Venereology; b Maharajgunj Medical Campus, Tribhuvan University Teaching Hospital; c Department of Dermatology and Venereology, Maharajgunj Medical Campus, Maharajgunj, Kathmandu, Nepal

**Keywords:** amoxicillin, ayurvedic, bullous erythema multiforme

## Abstract

**Introduction:**

Bullous erythema multiforme (BEM), an immune-mediated, acute condition, frequently includes erosion affecting the oral, genital, and/or ocular mucosa in addition to discrete target-like lesions on the skin. BEM has been linked to various factors, including infections, medications, malignancy, autoimmune disease, immunization, and radiation.

**Case presentation:**

Here, we report a case of a 38-year-old married woman who presented with symptoms of reddish-raised, fluid-filled and painful, nonpruritic lesions along with the swelling of bilateral hand and feet. This patient had a history of taking some unrecorded ayurvedic medication for bloating and abdominal pain in a background of antibiotic use before exhibiting the dermatological symptoms of BEM 2 days later. She was successfully managed with ampicillin and cloxacillin, acyclovir and prednisolone.

**Clinical discussion:**

A few incidence of BEM after the administration of amoxicillin has been reported, which precipitated only after consuming ayurvedic medication. BEM has a clinical diagnosis with biopsy rarely required. Here, the hypersensitivity reaction induced by the antibiotic itself or by altering the immune response to the concomitant consumed herbal medicine could explain the BEM.

**Conclusion:**

Physicians should note that amoxicillin can trigger BEM, regardless of its use with ayurvedic drugs. Antibiotics should be used with caution, especially in patients with a history of BEM.

## Introduction

Highlights

- Amoxicillin may cause bullous erythema multiforme (BEM); prompt withdrawal is critical.
- Prior BEM mandates cautious antibiotic use and monitoring.
- Hypersensitivity to the antibiotic or ayurvedic drug interaction may cause BEM.
- More research is needed for antibiotic–ayurvedic interaction in BEM.

Bullous erythema multiforme (BEM) is an immune-mediated mucocutaneous condition most commonly caused by infections and medication use^1^. Of the infections, 90% are triggered by the herpes simplex virus followed by *Mycoplasma pneumoniae*, particularly in children^2^. The most common medications that precipitate the disease are non-steroidal anti-inflammatory drugs (NSAIDs), sulfonamides, antiepileptics, and antibiotics^2^. It can also appear in the background of malignancy, autoimmune disease, radiation, immunization, and menstruation^3^. Although the precise incidence of BEM is unknown, it is estimated to range from 0.01 to 1%^4^. Younger adults are most likely to suffer from it, with a slight female predominance^5^. Erythema multiforme major is the name given to the type of condition in which the mucosa is affected; erythema multiforme minor is when the mucosa is unaffected^2^. It is a self-limiting condition, and the lesions most commonly resolve in 1–2 weeks, with the major variant taking longer. BEM has a clinical diagnosis with biopsy required rarely and is managed by treating symptoms with topical steroids or antihistamines and, if known, by treating the underlying cause^6^. We present the case of a 38-year-old female who developed BEM after consuming some undocumented herbal medicine while consuming amoxicillin for abdominal pain and bloating. She was successfully treated with ampicillin and cloxacillin, acyclovir, and steroids. This case report has been reported in line with the SCARE (Surgical CAse REport) Criteria^7^.

## Case presentation

A 38-year-old married female housewife by occupation presented with the chief complaint of reddish-raised, fluid-filled, and painful, nonpruritic lesions, along with swelling of the bilateral hand and feet. She started developing reddish-raised lesions over the dorsum of her right hand 3 days ago, which gradually spread to involve the bilateral dorsum of her hand and foot. A few similar lesions also appeared on her upper back and face within 2 days of the onset of hand lesions. She had a history of amoxicillin consumption for a sore throat. She also had a history of taking ayurvedic medication for abdominal pain and bloating on and off, which she took a few days after starting antibiotic medication, and that is when she started developing symptoms. There was no history of fever, cough, burning micturition, or insect bite. She did not provide a history of the application of topical herbal or cosmetic products. There is no history of similar illnesses in the past.

On admission, her general condition was fair [Glasgow Coma Scale (GCS) 13/15]. She was alert, conscious, cooperative, and well-oriented to time, place, and person. Her vital signs were stable and within normal limits except for low blood pressure (value: 90/60 mmHg). There was no pallor, icterus, lymphadenopathy, edema, dehydration, cyanosis, or clubbing. Her breathing sounds were normal with no added sounds. Her heart sounds S1/S2 were normal with no murmur. A soft tenderness over the left lumbar region was present with no organomegaly. Her higher mental functions, sensations, cranial nerves, and coordination were normal. The tone, power, and reflexes of her upper and lower limbs were normal. The rest of the systemic examination findings were regular. On cutaneous examination findings, there were multiple violaceous bullae over the erythematous base, the largest measuring ~3×4 cm and the smallest ~1×1 cm, with acral distribution mainly over the dorsum of bilateral hands and feet. A few papules and vesicles were scattered over the bilateral forearms, along with few erythematous plaques over the upper back. Her oral and genital mucosa were intact.

Investigations such as serology, urine culture and sensitivity (c/s), pus c/s, swab c/s, urine routine examination (RE), stool routine examination/microscopic examination, hematology, random blood sugar, renal function tests (RFTs), and liver function tests (LFTs) were performed. Hematology showed 13.3 g% hemoglobin with a packed cell volume of 38% and 311 000 platelet count. The total leukocyte count was 1140 cells/μl. The differential leukocyte count showed 80% neutrophils, 13% lymphocytes, 2% eosinophils, 5% monocytes, and nil basophils. The erythrocyte sedimentation rate was 90 with 2+ C-reactive protein. Urine RE showed trace albumin, 1–2 WBC (white blood cells), packed epithelial cells, and RBC (red blood cells) was nil. Urine c/s showed no growth. Serology was nonreactive. Pus c/s showed sterile pus. Swab c/s revealed Coagulase-negative *Staphylococci* sensitive to cloxacillin. RFTs showed 3.1 mmol/l urea, 45 μmol/l creatinine, 140 mEq/l sodium, 4 mEq/l potassium, 67 g/l protein, 36 g/l albumin, and total direct bilirubins of 7 and 1 μmol/l respectively. LFTs revealed that AST (aspartate aminotransferase), ALT (alanine aminotransferase), and ALP (alkaline phosphatase) were 13, 16, and 43 U/l, respectively. A diagnosis of BEM was made.

She was treated with ampicillin and cloxacillin, acyclovir and prednisolone. Daily dressing of the lesions was performed. Her lesions gradually started and improved, and she was clinically stable at the time of discharge. She was put on ampicillin and cloxacillin, acyclovir and mupirocin, and called up for follow-up after 2 weeks. The patient was completely well during her follow-up. She did not experience any side effects of the drugs, and the symptoms gradually disappeared.

## Discussion

BEM is an immune-mediated, acute condition that manifests as discrete target-like lesions on the skin and is frequently accompanied by erosion or bullae affecting the oral, genital, and/or ocular mucosa. The incidence of BEM is unknown, but it is estimated to range from 0.01 to 1%^4^. Drug-induced BEM is estimated to occur in less than 10% of cases^6^, although a higher rate has been found secondary to antibiotics (particularly penicillin) in children^5^. Drugs rarely cause BEM, but antiepileptic medications, NSAIDs, and antibiotics are frequently the culprits^6^. According to a database, 6.17% of cases of BEM were caused by taking paracetamol, and 2.06% of cases were caused by taking diclofenac sodium^8^. We suspected that amoxicillin could cause BEM and more so with relation to ayurvedic medicine consumption. In our case, the lesion manifested itself after the administration of the initial drug, amoxicillin. The main criteria for diagnosing BEM are clinical onset, a positive triggering factor, and clinical manifestation. The illness did not start until after taking some unreported ayurvedic medicine, and it continued even after stopping it as long as antibiotic use was continued. Because of the temporal relationship between drug intake and the onset of the lesion and the fact that the patient had not been exposed to any infection or food additives that might have triggered an allergic reaction, it was determined that the cause was amoxicillin. She did not come from a similar family history or had a similar past illness. She also did not mention any previous drug use. The patient believed the ayurvedic medicine was the source of her illness and discontinued it. She was psychologically affected because she had been isolated in her home due to her illness, far from community exposure. This incidence has been reported rarely, to our knowledge, in the scientific literature. Herb–drug interactions can occur when drugs and herbs are taken together because the former can mimic, amplify, or counteract the latter’s effects^9^. Tumor necrosis factor-alpha is expressed in drug-induced BEM instead of interferon-gamma. Drug metabolism is changed, directed toward the cytochrome p450-metabolite pathway, resulting in the production of reactive and toxic metabolites, and tissue damage is mainly caused by apoptosis^10^.

The most common way that BEM presents is with both cutaneous and mucosal lesions, although it can also only show cutaneous lesions. Rarely does BEM involve only the mucous membranes. Most patients with BEM experience oral involvement, which can affect them up to 70% of the time. The disorder is characterized by target lesions that have three distinct features: a dusky central area or blister, a dark red inflammatory zone surrounded by a pale ring of edema, and an erythematous halo on the lesion’s outermost edge^3^. Cutaneous lesions usually develop on the extensor surfaces of the acral extremities in a symmetrical pattern and then spread centripetally. Mucosal lesions commonly present as bullae, painful erosions, and/or diffuse areas of mucosal erythema and can affect the oral, ocular, and/or genital mucosa. In this case, there was no such lesion on the mucosa. The differentials include urticaria, Steven–Johnson syndrome, bullous pemphigoid, and paraneoplastic pemphigus, which can be distinguished from BEM by their course, presentation, distributions, and biopsies, if needed.

Identification and elimination of triggering factors are the cornerstones of BEM treatment. These lesions typically respond to corticosteroids; in the case of a minor lesion, topical steroids can be started, and systemic steroids can be used for severe conditions for a week while tapering the dose^11^. In our patient, she was prescribed a systemic steroid, prednisolone, for 2 weeks with antiviral therapy for 1 day. She was well-tolerated to given drugs and had no allergic reactions. The lesion healed completely after 2 weeks with no scars left.

## Conclusion

A physician needs to be mindful that amoxicillin can cause BEM with or without its association with ayurvedic drugs and that prompt withdrawal from these drugs is crucial. Antibiotic prescriptions in patients with prior BEM require careful monitoring and discretion. Further investigation on the antibiotic–ayurvedic medicine interaction regarding BEM is needed. However, a brief course of systemic prednisolone is extremely effective in controlling lesions, as supported by the striking response observed in our patient.

## Ethical approval

This study complies with the ethical principles outlined in the Declaration of Helsinki.

## Consent

Written informed consent was obtained from the patient for publication of this case report and accompanying images. A copy of the written consent is available for review by the Editor-in-Chief of this journal on request.

## Sources of funding

No funding has been received.

## Author contribution

S.S: involved in the treatment of the patient; B.K.S., S.K., A.Y., and N.P.: collected all the information, reports, and figures; reviewed the literature; and contributed to writing and editing the manuscript. All authors have read and approved the manuscript.

## Conflicts of interest disclosure

The authors report no conflicts of interest in this work.

## Research registration unique identifying number (UIN)

It’s a case report and might not need registration. If it does, kindly let us know.

## Guarantor

Bikash Kumar Shah.

## Data availability statement

Yes, they are publicly available.

## Provenance and peer review

No, it was not invited.
